# The importance of distinguishing between the odds ratio and the incidence rate ratio in GWAS

**DOI:** 10.1186/s12881-015-0210-1

**Published:** 2015-08-30

**Authors:** Berit Lindum Waltoft, Carsten Bøcker Pedersen, Mette Nyegaard, Asger Hobolth

**Affiliations:** National Center for Register-based Research, Department of Economics and Business Economics, Aarhus University, Fuglesangs allé 4 room K10, 8210 Aarhus V, Denmark; The Lundbeck Foundation Initiative for Integrative Psychiatric Research, iPSYCH, Aarhus, Denmark; Bioinformatics Research Center, Aarhus University, C.F. Møllers Allé 8, 8000 Aarhus C, Denmark; The Centre for Integrated Register-based Research, Aarhus University, CIRRAU, Arhus University, Aarhus, Denmark; Department of Biomedicine, Aarhus University, Vennelyst Boulevard 4, 8000 Aarhus C, Denmark

**Keywords:** Genome wide association study, Study design, Matched case-control study, Competing risk, Logistic regression, Conditional logistic regression, Rare disease assumption

## Abstract

**Background:**

In recent years, genome wide association studies have identified many genetic variants that are consistently associated with common complex diseases, but the amount of heritability explained by these risk alleles is still low. Part of the missing heritability may be due to genetic heterogeneity and small sample sizes, but non-optimal study designs in many genome wide association studies may also have contributed to the failure of identifying gene variants causing a predisposition to disease. The normally used odds ratio from a classical case-control study measures the association between genotype and *being* diseased. In comparison, under incidence density sampling, the incidence rate ratio measures the association between genotype and *becoming* diseased. We estimate the differences between the odds ratio and the incidence rate ratio under the presence of events precluding the disease of interest. Such events may arise due to pleiotropy and are known as competing events. In addition, we investigate how these differences impact the association test.

**Methods:**

We simulate life spans of individuals whose gene variants are subject to competing events. To estimate the association between genotype and disease, we applied classical case-control studies and incidence density sampling.

**Results:**

We find significant numerical differences between the odds ratio and the incidence rate ratio when the fact that gene variant may be associated with competing events, e.g. lifetime, is ignored. The only scenario showing little or no difference is an association with a rare disease and no other present associations. Furthermore, we find that *p*-values for association tests differed between the two study designs.

**Conclusions:**

If the interest is on the aetiology of the disease, a design based on incidence density sampling provides the preferred interpretation of the estimate. Under a classical case-control design and in the presence of competing events, the change in *p*-values in the association test may lead to false positive findings and, more importantly, false negative findings. The ranking of the SNPs according to *p*-values may differ between the two study designs.

**Electronic supplementary material:**

The online version of this article (doi:10.1186/s12881-015-0210-1) contains supplementary material, which is available to authorized users.

## Background

The genome wide association study (GWAS) is a powerful tool to associate genetic variation with disease, and thousands of associations have been established and replicated [1]. Through the development of new methodology, the results from the GWAS's have been useful in other settings. One example is in health-related epidemiology studies (e.g. Ligthart et. al. [2] and Simonson et. al [3]). Here, the estimated odds ratio from the GWAS was used as a proxy for genetic variation through the polygenic score [4, 5]. The estimated value of the odds ratio is most often based on a number of unrelated cases and controls, and estimates the association between genetic variation and the prevalence of disease.

However, events precluding the disease of interest may play an undesired and crucial role when sampling cases and controls for a GWAS. If some of the genotyped SNPs are correlated with a disease but also with other events precluding this e.g. early death, a carefully chosen study design is needed to identify true associations with the aetiology of the disease. Precluding events may arise due to pleiotropy and are often referred to as competing events [6, 7]. These possible (and most often hidden) associations can be taken into account using incidence density sampling matching on age. This design measures the association between SNPs and acquisition of the disease and the effect size is expressed as the incidence rate ratio. Whether or not to take competing events into account in a GWAS depends on the scientific question. The classical case-control study provides a measure of the association between genotype and *being* diseased, whereas the incidence density sampled study associates genotype with *becoming* diseased.

In this paper, we explain two widely used measures of association: the odds ratio (OR) and the incidence rate ratio (IRR) [8]. We explain, using an analogy with leaves falling from a tree, the different interpretations of the prevalence and the incidence rate, and how competing events can affect the interpretation of disease associations. In order to understand and quantify in detail the difference between the OR and the IRR of disease we consider two sampling methods for controls: incidence density sampling (or matching) and unrelated case-control sampling. We use simulations to quantify the difference between the association estimates from the two study designs under different effects of competing events.

We find considerable numerical differences between the OR and the IRR. The differences are reflected in a change in the *p*-values of the association tests. Our findings have significant implications for GWASs, since they point out the risk of serious false negative associations caused by competing events.

## Methods

### Prevalence vs. Incidence rate

To illustrate the prevalence, the incidence rate, and the effect of competing events, one may imagine a large tree in a park. The tree has two kinds of leaves: heavy leaves and light leaves. Consider a dominant model, corresponding to a population of individuals with two levels of one exposure (presence or absence of risk allele). When leaves fall from the tree they can either fall onto the sticky soil or onto the non-sticky concrete. Leaves falling on the concrete will eventually move to the soil. Falling onto the soil will decompose the leaf, corresponding to dying, and falling onto the concrete corresponds to becoming sick. The healthy leaves are still hanging on the tree. The heavy leaves will not be carried by the wind as easily as the light leaves, and therefore the heavy leaves have a tendency to fall on the soil under the tree more often than the light leaves, and the light leaves have a tendency to fall onto the concrete. Among leaves falling on the concrete, the heavy leaves have a tendency to stay longer on the concrete, before they are blown onto the soil, than the light leaves.

The number of leaves falling from the tree onto the concrete per time unit corresponds to the incidence rate of disease i.e., the light leaves have a higher incidence rate than the heavy leaves. The number of leaves falling onto the soil per time unit corresponds to the incidence rate of death i.e. the light leaves have a smaller incidence rate of death than the heavy leaves. The prevalence of disease is the fraction of leaves on the concrete at a given time period compared to the total number of leaves on the tree and on the concrete. The odds of disease is the number of leaves on the concrete at a given time point compared to the number of leaves on the tree at the same time, i.e. the prevalence of disease divided by one minus the prevalence of disease.

The OR, normally calculated in a GWAS, is a function of the prevalence. It is the ratio between the odds of disease for light leaves compared to the odds of disease for heavy leaves. It measures the association between the exposure and *being* diseased. The IRR is the fraction of the incidence rate for light leaves vs. the incidence rate for heavy leaves, and it measures the association between the exposure and *becoming* diseased. The OR depends on the incidence rate of leaves falling onto the concrete and the incidence rate of leaves moving onto the soil both from the tree and from the concrete. Because of these dependencies SNPs may be associated with a disease through the OR, but not through the IRR and vice versa.

### Measures of association

When the exposure or the outcome is not available for all individuals in a population, it is impossible to set up a full cohort study, and different types of sampling are used. Most often the measure of disease occurrence is calculated as the number of cases divided by the number of controls, maybe for different exposure groups. The measure of association between exposure and disease is then calculated as the ratio between the measures of disease occurrence in two different groups of the exposure [9].

When using a case-control sample to estimate the association between an exposure and a disease of interest, the interpretation of the association depends on the procedure used to select controls. Two different methods are normally used to select controls according to the onset of disease: the unrelated case-control sampling and the incidence density sampling.

**Fig. 1:**
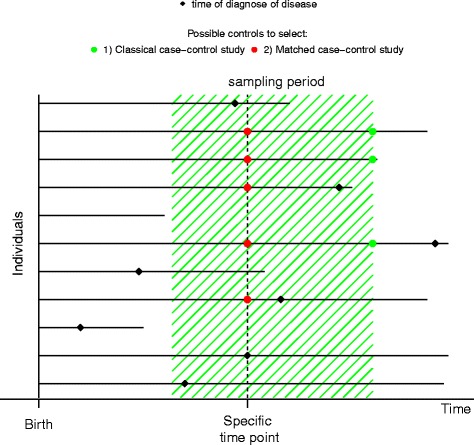
Longitudinal data. Each horizontal line indicates an individual’s lifespan. The black filled diamonds indicate that the given individuals become diseased. The green hatched area indicates the sampling period of the case-control study. The green dots indicate all possible controls in a classical case-control study (design 1). The red dots indicate all possible controls for incidence density sampling matched to the selected case (design 2)

The two sampling methods are displayed in Fig. 1. Each horizontal line in the figure corresponds to a lifespan of an individual. The individual is born when the line starts and dies when the line ends. A black diamond marks time of onset of the disease of interest. The green hatched area corresponds to a sampling period for capturing cases and controls for a classical case-control study. The green and red dots mark individuals, who are eligible controls for the two different sampling methods described below.

1. In the classical case-control study all cases are chosen within a certain time period. The sampling time is specified by the investigators. The controls are chosen uniformly at random among those who have not experienced the event after the sampling period (green dots in Fig. 1). The number of cases proportional to the number of controls estimates the odds of disease, and the OR is estimated as the fraction between two odds. The measure of disease occurrence in this setup is the odds, which is a function of the prevalence [10].
2. Using incidence density sampling controls are sampled longitudinally in time. The controls are chosen uniformly at random among persons at risk of developing the disease at the time when a case appears (red dots in Fig. 1), i.e., each person who develops the disease of interest during the sampling period has their own set of potential controls. This is also called time-matching [11]. The measure of disease can be interpreted as the probability of becoming diseased in the next time increment, i.e., the incidence rate of disease. The IRR is the association measure [12]. If the data support an assumption of constant instantaneous probability of disease in small intervals of time, then the controls may instead be chosen within a small time window (and not at specific time points). Contrary to the OR, the IRR is not dependent on the prevalence of the disease.

### Simulation study

To quantify the differences between the OR and the IRR, if any, we simulate a longitudinal study with one exposure and two events; disease and death. The longitudinal study includes 10,000 individuals. From this study we generate two different case-control studies using the sampling designs described above to collect controls: the classical case-control study and the matched case-control study using incidence density sampling. In detail we do the following:

For all 10,000 individuals in the simulated data we generate the number of minor alleles under the assumption of Hardy-Weinberg equilibrium and a minor allele frequency (MAF) of 0.1, 0.25 and 0.5.

In order to generate a longitudinal data source with one exposure, we use the algorithm discussed by Beyersmann et al. [13] using two events: disease and death. An individual’s waiting time to any event is generated as a function of the total incidence rate of the two events over time. At the given time point of an event, the probability of disease is the proportion between the incidence rate of disease and the total incidence rate. Individuals still alive at age 90 are censored.

**Fig. 2:**
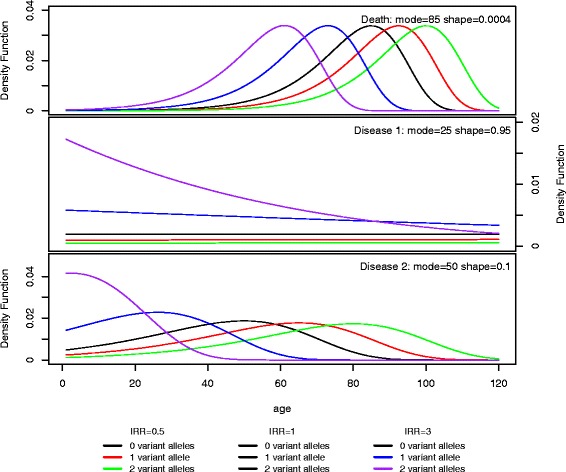
The density of the three outcomes. The densities of the three types of outcome; death and two different diseases. The black solid line in subfigure A is the density function of a Gompertz distribution with density mode at age 85 and a shape parameter of 0.0004, i.e. the overall baseline. This is the likelihood of dying given an individual’s age. The black solid line in subfigures for Disease 1 and Disease 2 is the density function of a Gompertz distribution with density mode at age 25 and a shape parameter of 0.95 and mode at age 55 and shape parameter 0.1 respectively. This is the likelihood of getting diseased given an individual’s age. The coloured lines correspond to the likelihood of dying or developing the disease for different genotypes and different associations between the SNP and death

We use a Cox proportional hazard model where the baseline hazards follow a Gompertz distribution [14] for both events. The baseline for the incidence of dying follows a Gompertz distribution with mode 85 and shape parameter 0.0004. The densities for different effects of the number of minor alleles on the IRR of death are shown in Fig. 2. Two different parameter settings are used for the baseline of disease: one with mode 25 and shape parameter 0.95, and one with mode 50 and shape parameter 0.1. Figure 2 shows the densities for the two parameter settings for different effects of the number of minor alleles on the IRR. The black lines in Fig. 2 correspond to the overall baseline density. We multiply the incidence rate of disease by a constant (less than 1) in order to scale the life-time risk of disease [15]. Different values of the constant are used to consider common versus rare diseases; the smaller the constant the rarer the disease. We assume a log linear proportional effect of the number of minor alleles on the incidence rate of disease and death, with different incidence rate ratios of 0.5, 1.0, 1.1, 1.2, 1.5, 1.7, 2.0 and 3.0 for disease and 0.5, 0.75, 0.9, 1.0, 1.1, 1.2, 1.5, 1.7, 2.0, and 3.0 for death.

From the simulated longitudinal data we generate two epidemiological study designs to estimate the association between the number of minor alleles and disease. For the incidence density sampling, we choose 5 individuals uniformly at random among the persons not diseased and not dead at the age when an individual becomes diseased. This result in a 1: 5 individually time matched nested case-control study, where cases and controls are matched on age. The association between the number of minor alleles and disease is estimated using conditional logistic regression (see chapter 7 in [16]).

The cases in the unrelated case-control sampling are individuals diseased before the age of 90. The controls are chosen among all persons not diseased before the age of 90, that is, the sampling period is from birth to age 90 (see Fig. 1). If the number of possible controls is larger than five times the number of cases, the controls are chosen uniformly at random at a 1:5 ratio between cases and controls. We estimate the association between the number of minor alleles and disease in the case-control sample using logistic regression (see chapter 6 in [16]). Additionally, we simulate an age covariate for each individual in the classical case-control study. For controls, the age is sampled from a uniform distribution on the interval form birth to end of follow-up. For cases the covariate is the age at which the individual is diagnosed. We then estimate the association between the number of minor alleles and disease adjusted for a linear age trend in the classical case-control study.

In total we generate 1000 longitudinal data sources for each configuration of MAF, IRR of disease, IRR of death, the rarity of the disease and the two parameter settings of the baseline rate of disease. The measure of association in a given analysis is estimated as the average of the 1000 replications. The null hypothesis is no association between the number of minor alleles and disease prevalence in the classical case-control study, and is no association between minor alleles and disease incidence in the incidence density sampled study. The *p*-value of this hypothesis is estimated as 2 times the proportion of estimates less than 0 if the measure of association is larger than 0, and it is estimated as 2 times 1 minus the proportion of estimates less than 0 if the measure of association is less than or equal to 0. The proportion less than 0 are calculated based on the 1000 replicates (see Additional file 1: R code for programming details).

## Results

The estimate from the incidence density sampling of controls is not numerically different from the true IRR determined by the simulation study irrespective of the baseline of disease, the IRR of death and the MAF (results not shown).

**Fig. 3:**
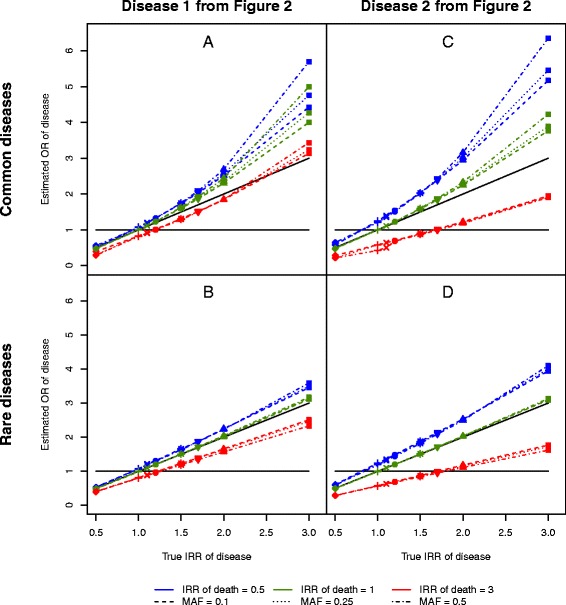
The estimated associations. Four scenarios are displayed: two different diseases (one rare and one common) and two different parameter settings for the likelihood of developing the disease. The probability of being diseased at age 90, i.e. the cumulative incidence rate of disease, is as follows for the four figures. **a**: 21.8 %, **b**: 2.3 %, **c**: 22.9 % and **d**: 2.6 %. Each subfigure presents the estimated association between the number of minor alleles and disease for different values of the IRR of death and the MAF. The different colours indicate different values of the IRR of death and the different line types indicate the different values of the MAF. The sloped, black solid identity line indicates no bias and the horizontal line at OR of disease = 1 indicates no association

For the classical case-control study, Fig. 3 shows the estimated associations between the disease and the number of minor alleles for each parameter setting of the baseline rate of disease and for a common and a rare disease (the IRR for death equals 0.5, 1.0 and 3.0. The remaining results are given in the online Additional file 2: Figure S2, including corresponding figures for the age-adjusted model in Figure S1 and Figure S3). In each subfigure one estimate is shown for different settings of the true IRR of disease, the IRR of death and the MAF. The probability of disease at age 90, i.e. the cumulative incidence at age 90, is approximately 22 % for the common disease and 2.5 % for the rare disease (see the legend of Fig. 3). The black solid diagonal line indicates no numerical differences between the estimated OR and the true IRR, whereas the black solid horizontal line at 1 indicates no association according to the estimated OR.

**Fig. 4:**
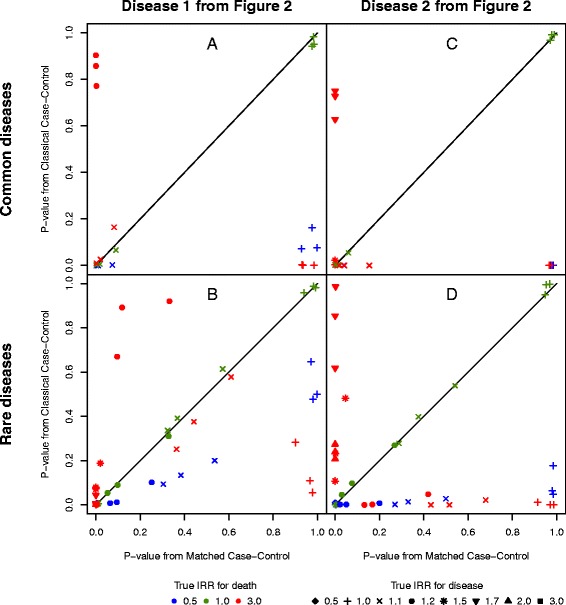
The p-value for the associations. Four scenarios are displayed: two different diseases (one rare and one common) and two different parameter settings for the likelihood of developing the disease as in Fig. 3. The probability of being diseased at age 90, i.e. the cumulative incidence rate of disease, is as follows for the four figures. a: 21.8 %, b: 2.3 %, c: 22.9 % and d: 2.6 %. Each subfigure is a QQ-plot of the two *p*-values of the hypothesis of no association, one generated under the classical case-control study and the other generated under the matched case-control study for each of the true IRR of disease and the RR of death. The results from the parameter configurations where both *p*-values equal 0 are not displayed. The black solid line indicates the diagonal

Figure 4 shows the *p*-values for the null hypothesis of no association for the two different sampling methods and for different values of the true IRR of disease and the IRR of death. The three identical colour-symbol combinations correspond to the different MAF. The scenarios and symbols in Fig. 4 are similar to those in Fig. 3. The *p*-values from the matched case-control study are plotted on the horizontal axis and the *p*-values from the classical case-control study are plotted on the vertical axis. For each subfigure we see false positive findings below the diagonal and false negative findings above the diagonal. Scenarios where both *p*-values equal zero are not plotted.

If the number of minor alleles is associated with longevity, i.e. the IRR for death is less than 1, then the estimated OR is larger than the true IRR of disease, irrespective of the rarity of the disease, the baseline rate of disease and the MAF. The differences become larger when the value of the true IRR for disease is large, and the differences are larger for common diseases than for rare diseases (see blue symbols in Fig. 3). The larger estimates are reflected in false positive findings. In the case of no true association (the true IRR for disease equals 1) the estimated OR is significantly different from 1 for common diseases (see blue symbols in Fig. 4). Generally, the *p*-values from the classical case-control study are smaller than the *p*-values from the matched case-control study.

When there is no association between the number of minor alleles and lifetime, i.e. the IRR of death is 1, the estimated OR is larger than the true IRR for common diseases when the true IRR for disease is larger than 1 (green symbols in Fig. 3a and c). For the rare diseases, there is no difference between the estimated OR and the true IRR of disease (green symbols in Fig. 3b and d). The *p*-values from the classical case-control study and from the matched case-control study are similar, thus all green symbols in Fig. 4 follow the solid black line.

When the true IRR for death is larger than 1, the estimated OR is smaller than the true IRR for disease (red symbols in Fig. 3). In Fig. 3a the estimated OR is larger than the true IRR for death, for large true values. The smaller estimated OR gives rise to false positive associations (see + in Fig. 4a and d). False negative associations occurred when the estimated OR is close to 1 and the true IRR is larger than 1, e.g. when the IRR of the true association with disease is 1.7 (see in Fig. 3 and 4c or d). If the true IRR equals 1.1 (x) Fig. 4c reveals a positive test for association. The same symbols, x, on Fig. 3c shows the estimated OR is significantly smaller than 1, whereas the true association is an increase in the incidence rate of disease (the true IRR equals 1.1).

Including age in the model introduces slightly smaller differences for the estimates in scenario A and B in Fig. 3 (see Additional file 2: Figure S1). For scenario C there are smaller differences when the IRR for death is larger than 1, but a slightly larger difference if the IRR for death is smaller than or equal to 1. The largest effect of the adjustment is seen in scenario D, where the introduction of age as a covariate considerably reduce the differences irrespective of the value of the IRR of death.The differences between the estimated OR and the true IRR for disease, for different values of the MAF and the IRR for death, are displayed in Additional figure 2: Figure S4 and Figure S5 (age adjusted). The relative differences between the two are displayed in Additional figure 2: Figure S6 and Figure S7 (age adjusted).

**Fig. 5:**
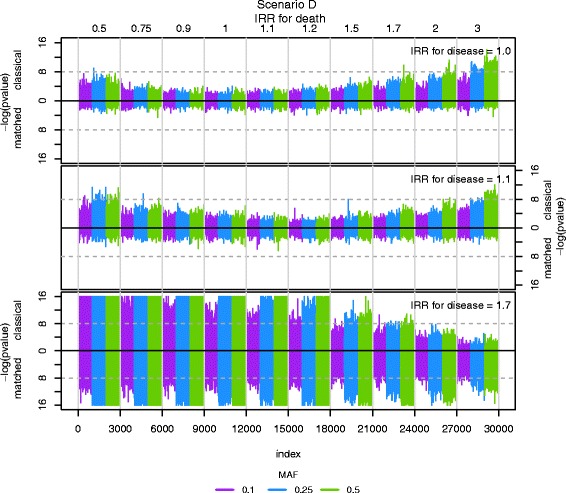
Comparison of *p*-values from the simulations. The scenario D (from Fig. 3 and Fig. 4) is displayed. The three subfigures represent the IRR for disease equal to 1.0, 1.1 and 1.7 respectively. Each subfigure is two Manhattan plots consisting of –log transformed *p*-values from all 30,000 simulations. Above the solid black line at 0 is the –log transformed *p*-values from the classical case-control study and below is the –log transformed *p*-values from the incidence density sampling. The different colours indicate a parameter change in the MAF and the light grey vertical lines indicate the change in the IRR of death. The horizontal dashed lines indicate the genome-wide significance level of 5 · 10^−8^

For scenario D in Figs. 3 and 4, Fig. 5 displays the comparison of the *p*-values for all simulated results where the IRR for disease is 1.0, 1.1 and 1.7 (scenarios A, B and C are displayed in the online Additional file 2: Figure S8, S9 and S10). The –log transformed *p*-values above the black horizontal line represent the classical case-control study, whereas those below the horizontal line originate from the matched case-control study. The dashed lines indicate a genome-wide significance level of 5 · 10^−8^. For clarity, all *p*-values lower than 10^−16^ are truncated. For large and small values of the IRR for death and for the IRR for death equal to 1.0 and 1.1, the *p*-values from the classical case-control study are lower than those from the incidence density sampled study, to the extent of genome-wide significance. When the IRR for disease equals 1.7, the *p*-values from the case-control study decrease more rapidly than those from the incidence density sampled study. It falls below the genome-wide significance level.

## Discussion

For each configuration of the rarity of the disease, the parameter setting of the baseline rate of disease, the MAF, and the IRR of disease and death, we simulated 1000 estimates of the odds ratio of disease. For each configuration, the standardized estimates of the OR for disease are, according to the central limit theorem (e.g. [17]), standard normal distributed.

There is no difference between the estimated and true association of disease and the number of minor alleles using incidence density sampling. In the classical case-control study we observe differences between the estimated OR and the true IRR in both directions, depending on the IRR for death. We find a larger estimated OR if there is an association between the number of minor alleles and longevity, i.e., the IRR for death is less than 1. If the association between the number of minor alleles and death is larger than 1, the estimated OR is smaller. However, these differences are small when the effect of the competing association is small. Small effects of competing events (below 1.3) are expected in a GWAS, since no evidence of large effects is found so far. When there is no association between the number of minor alleles and competing events we find a larger estimated OR if the disease is common, whereas there are no differences if the disease is rare. Others find similar results, e.g. Fradin et al. [18] and Wang et al. [19].

The fact that we do not find any differences if the SNP is not associated with death and the disease is rare, is known as the “rare disease assumption”, i.e. the OR is asymptotically equal to the IRR if the disease is rare [9, 20]. Notice that the “rare disease assumption” is compromised if there is an association between the SNP and competing events. An inference on the probability of disease may be biased due to competing events, even in a longitudinal study. Fine and Gray [21] developed a method allowing association testing of the effect of a SNP on the probability of disease.

From Fig. 5 we identify differences in the *p*-values both towards false positive hypothesis and false negative hypothesis. These differences are due partly to the larger or smaller estimated OR and to the effect of the total number of cases on the statistical power of the studies. *P*-value differences may introduce different rankings of SNPs between the two study designs. The ranking of the SNPs in a GWAS using a classical case-control study gives a list of SNPs most associated with the prevalence of disease, whereas the incidence density sampled study ranks SNPs according to the impact of the SNP on the aetiology of the disease. If the aim of the association study is to identify genetic markers involved in the genetic aetiology of the disease, then incidence density sampling allows for the correct interpretation of the estimates.

When a summary of the OR estimates from a classical case-control study is used as a proxy for genetic variation, e.g. in the polygenic score, the differences between the estimated OR and the IRR are aggregated into one measure. It is unknown if and to what extent this aggregation affects the validity of the approximation. The possible change in rankings can lead to summary statistics based on false positive SNPs. The approximation may be affected more, though, by the exclusion of false negative SNPs. The impact of ignoring the false negative SNPs is unknown and request further research.

In studies using a well-documented dichotomous confounder, the incidence density sampled study has higher power than the classical case-control study [22, 23]. However, in practice this may not always be the case. If a predefined incidence density sampled study cannot collect all needed information on both cases and controls the matching may be broken. In a 1:1 matched design this means a loss of both the case and the control in the analysis, reducing the power due to loss of information [24]. In the classical case-control study, loss of information for a single subject will not imply a loss of information for other subjects in the study.

The presence of competing events will decrease the number of cases observed, and will decrease the number of available matched sets in the incidence density sampling. The power of a matched study depends on the number of sets, and therefore the power is dependent on the effect of the competing event. The decreasing number of cases will also affect the power of a classical case-control study. This effect is, however, assumed to be smaller than under incidence density sampling [25]. The literature in this field of research is sparse, and many questions are still to be answered, especially when including quantitative measures and more complex models.

Planning an incidence density sampled study requires information on established confounders, and these are not always present. Under incidence density sampling, matching on non-informative confounders may in some situations introduce data overfitting, and thereby bias on both the estimated IRR and on the standard error [25]. Even matching on an established confounder introduces bias, if this confounder is correlated with one of the unmatched confounders in the study.

When matching on age, the birthdays of all individuals, and the date or period of which the case become diagnosed are particularly important. This information may be hard to retrieve using surveys or other self-reporting sampling techniques. Hence, the incidence density sampled study is difficult to implement in countries without access to nationwide population-based registers.

The simulations in this study are a simplified example of a GWAS only including one time scale and one genetic marker. Since risk of death increases with increasing age, age is an important factor to consider for matching. In other diseases, calendar time has a major effect due to changes in diagnostic systems, changes in politics etc. In such cases, one should consider which of the two time-scales is the most important if the possibility of using both is limited. One should also consider other factors for matching that have a large impact on the risk of disease, e.g. gender, place of birth or work environment.

When selecting controls in the incidence density sampling, the controls should be chosen at random among persons still at risk at the time of disease onset of the case. The controls are a random sample of the population at that specific time point. Leaving out controls that later become cases or never experience other diseases, will cause bias to the estimated IRR [11, 26].

In comparison with the analogy of the tree, this study does not take into account the association between leaf type and the likelihood of falling onto the soil, among the leaves on the concrete. Including this association will make the interpretation of the estimated OR and the effect on the *p*-values more complex, since this association will be reflected in the estimated OR. This association will however most likely be present in any population. For simplicity, we have also chosen to consider death as the only competing event. One should keep in mind that other events exist, such as emigration, which, for some age groups, are more likely than death [27]. The difference between the OR and the IRR is affected by an aggregation of all possible competing events. We urge the genetic community to carefully consider the specific research question and hence the sampling for any GWAS.

## Conclusions

Competing events are universally related to human life precluding individuals from specific outcomes. For the identification of aetiological factors related to disease, interest is in the risk of becoming diseased. Using a classical case-control study to estimate the association between risk of becoming diseased and genetic variants may bias the association estimate and the *p*-value of the association test. The incidence density sampled study will provide the correct interpretation of the estimated association and the proper ranking of SNPs for further analysis.

## Additional files

Additional file 1:
**The R code for simulations.** (ZIP 2.06 kb) Additional file 2:
**Supplementary Figures S1–S10 with captions.** (PDF 3.29 mb)
