# Supplementary material for: The importance of distinguishing between the odds ratio and the incidence rate ratio in GWAS
Source: BMC Med Genet. 2015 Aug 30;16:71. doi: 10.1186/s12881-015-0210-1 (PMC4593225; doi:10.1186/s12881-015-0210-1)
Supplement: Additional file 2: — Supplementary Figures S1–S10 with captions. (PDF 3.29 mb) [file 12881_2015_210_MOESM2_ESM.pdf]

Supplementary Figures

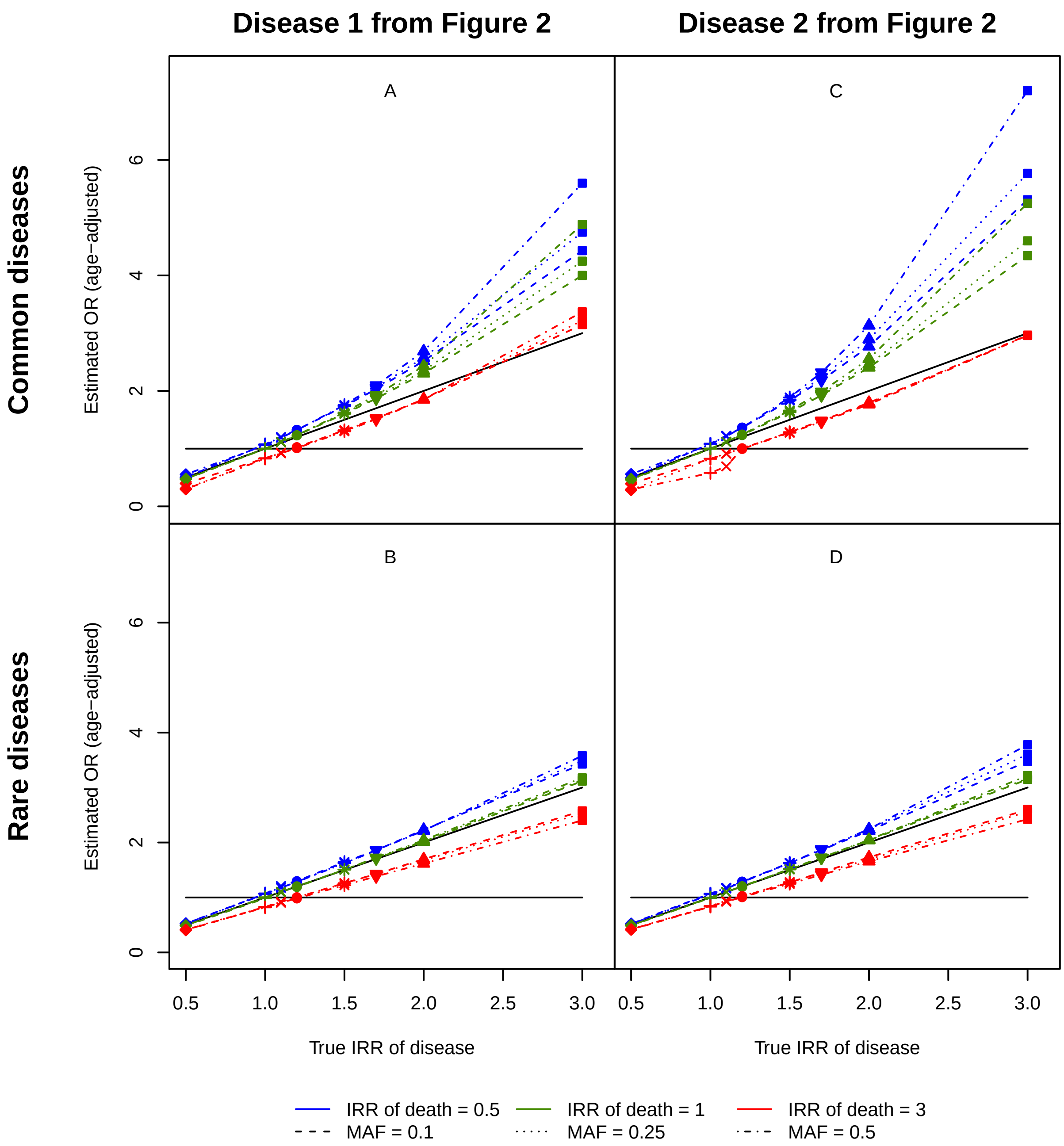

Figure S1: The estimated association when adjusting for age. Four scenarios are displayed as in Figure 3: two different diseases (one rare and one common) and two different parameter settings for the likelihood of becoming diseased. The probability of being diseased at age 90, i.e. the cumulative incidence rate of disease, is as follows for the four subfigures: A: 21.8%, B: 2.3%, C: 22.9% and D: 2.6%. Each subfigure presents the estimated association between the number of minor alleles and disease for different values of the IRR of death and the MAF (adjusted for age using a linear trend). The different colours indicate different values of the IRR of death, and the different line types indicate the different values of the MAF. The sloped, black solid line indicates no bias and the horizontal line at estimated OR = 1 indicates no association.

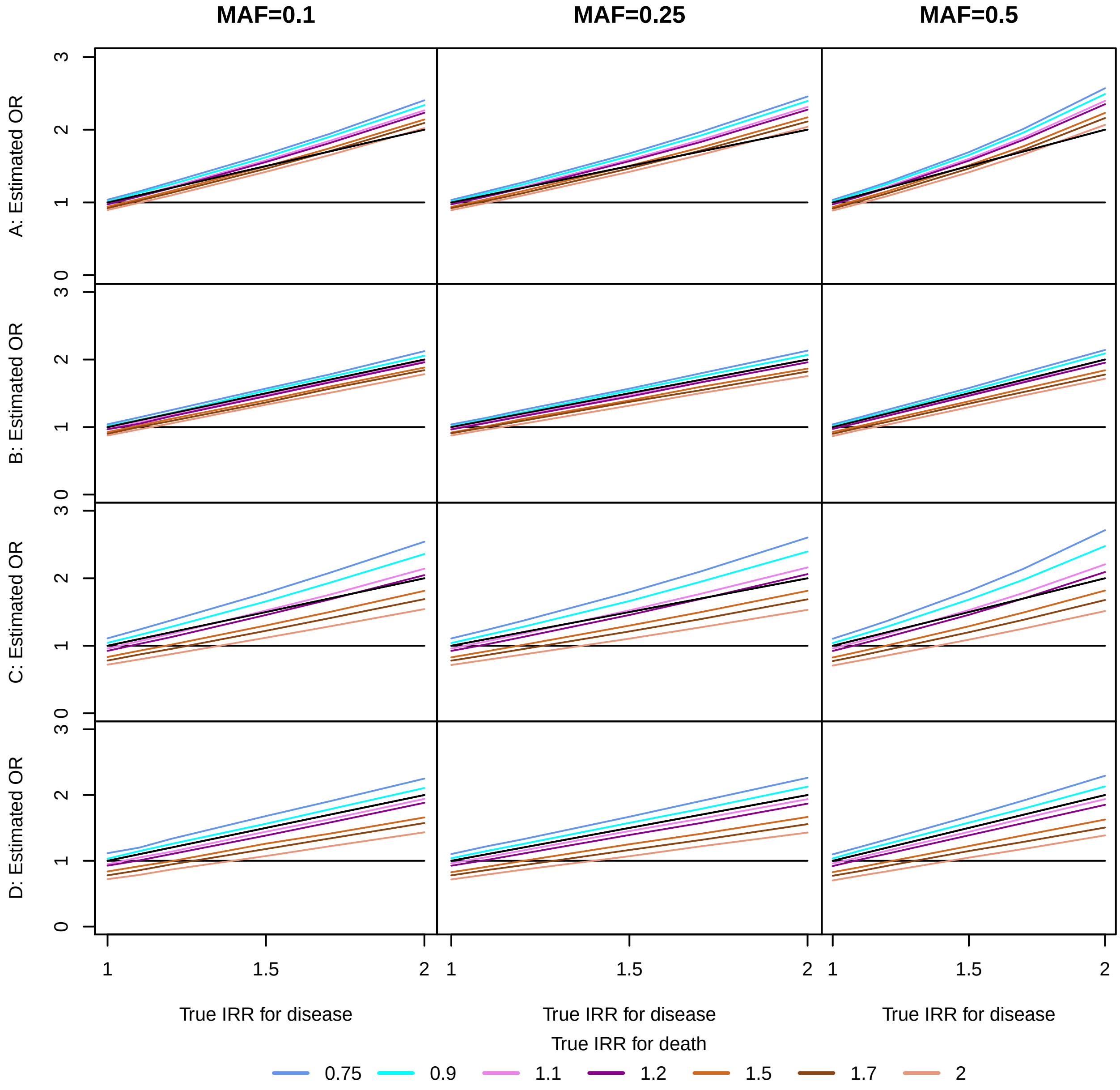

Figure S2: The estimated association for other IRR for death. Four scenarios are displayed as in Figure 3 and Figure S1. Each subfigure presents the estimated association between the number of minor alleles and disease for different values of the IRR of death and the MAF. The different colours indicate different values of the IRR of death. The sloped, black solid line indicates no bias and the horizontal line at estimated OR =1 indicates no association.

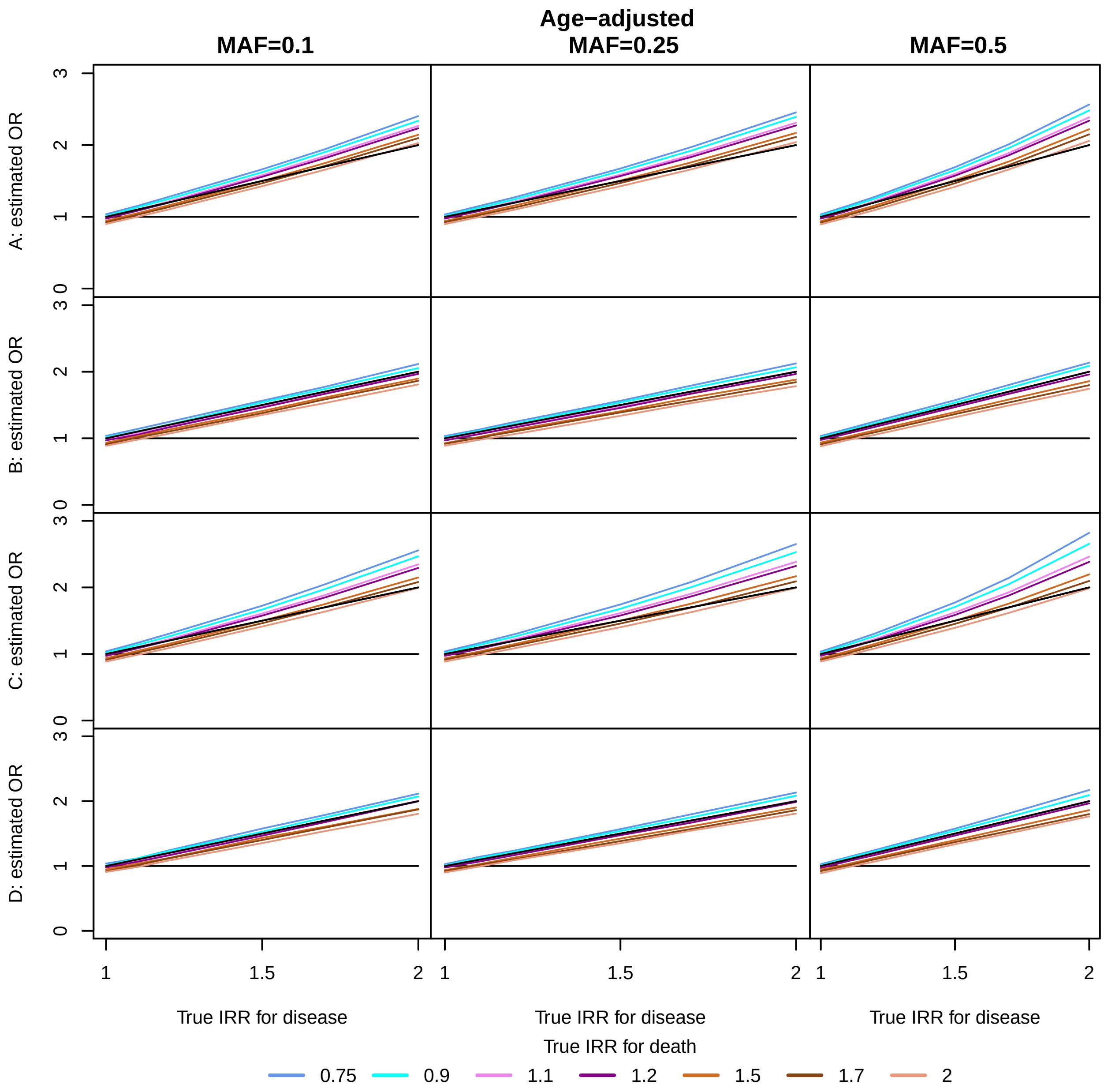

Figure S3: The estimated association for other IRR for death when adjusting for age. Four scenarios are displayed as in Figure 3 and Figure S1. Each subfigure presents the estimated association between the number of minor alleles and disease for different values of the IRR of death and the MAF (adjusted for age using a linear trend). The different colours indicate different values of the IRR of death. The sloped, black solid line indicates no bias and the horizontal line at estimated OR =1 indicates no association.

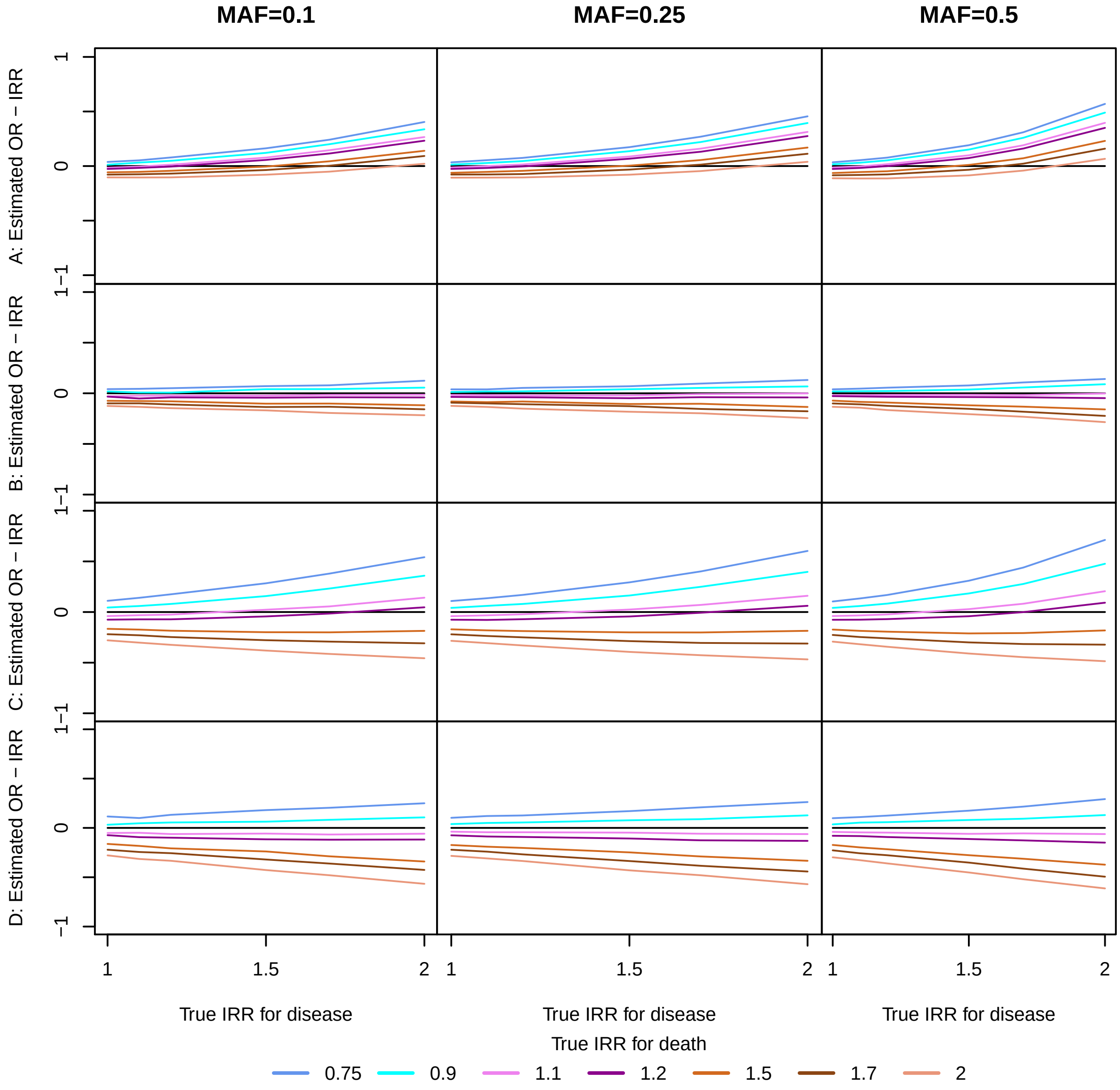

Figure S4: The differences in estimates. Four scenarios are displayed as in Figure 3 and Figure S1. Each subfigure presents the differences in the estimate of the association between the number of minor alleles and disease for different values of the IRR of death and the MAF. The difference is calculated as the estimated association minus the true association. The different colours indicate different values for the IRR of death. The black solid horizontal line indicates no bias.

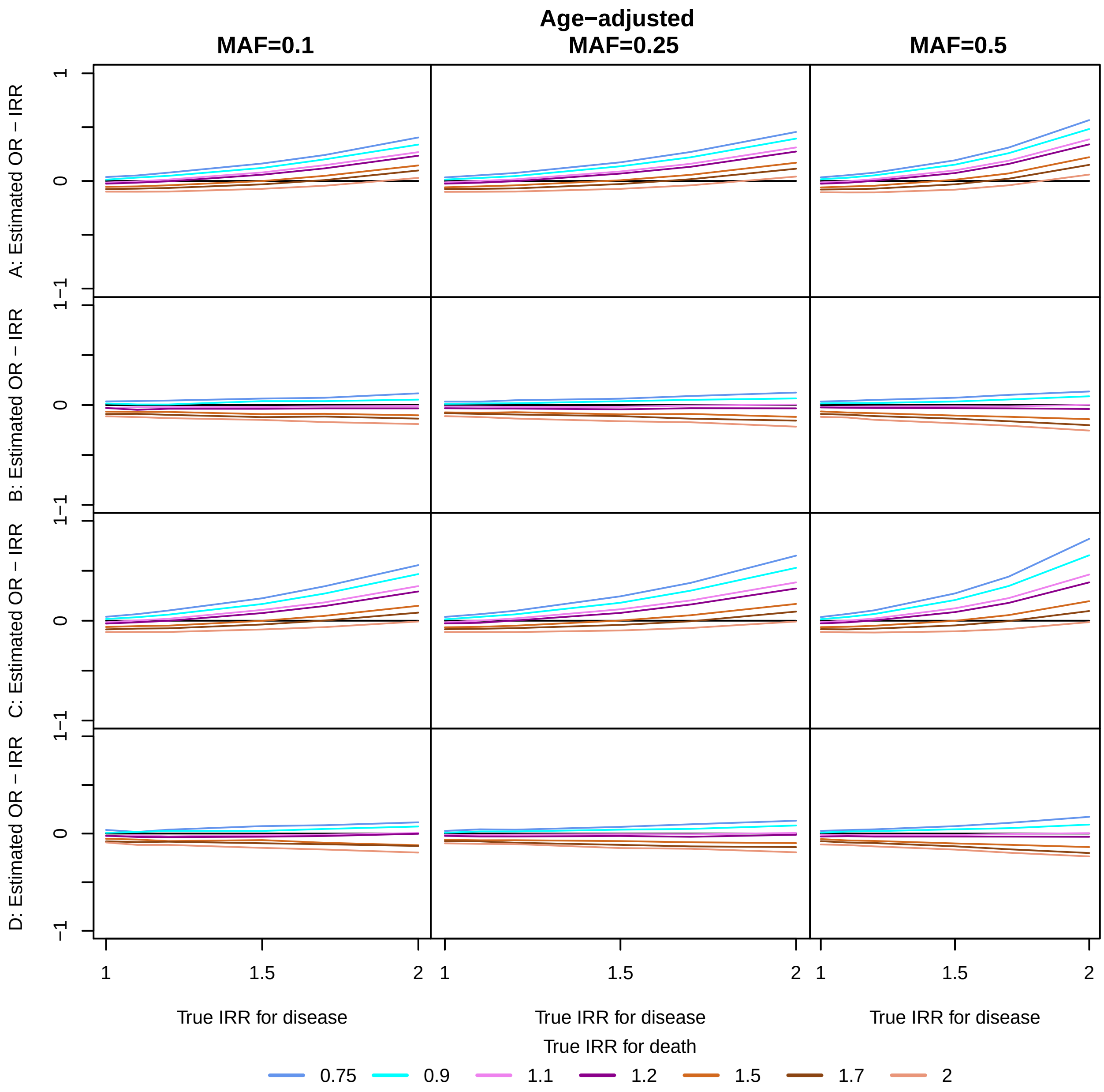

Figure S5: The differences in estimates when adjusting for age. Four scenarios are displayed as in Figure 3 and Figure S1. Each subfigure presents the differences in the estimation of the association between the number of minor alleles and disease for different values of the IRR of death and the MAF (adjusted for age using a linear trend). The difference is calculated as the estimated association minus the true association. The different colours indicate different values for the IRR of death. The black solid horizontal line indicates no bias.

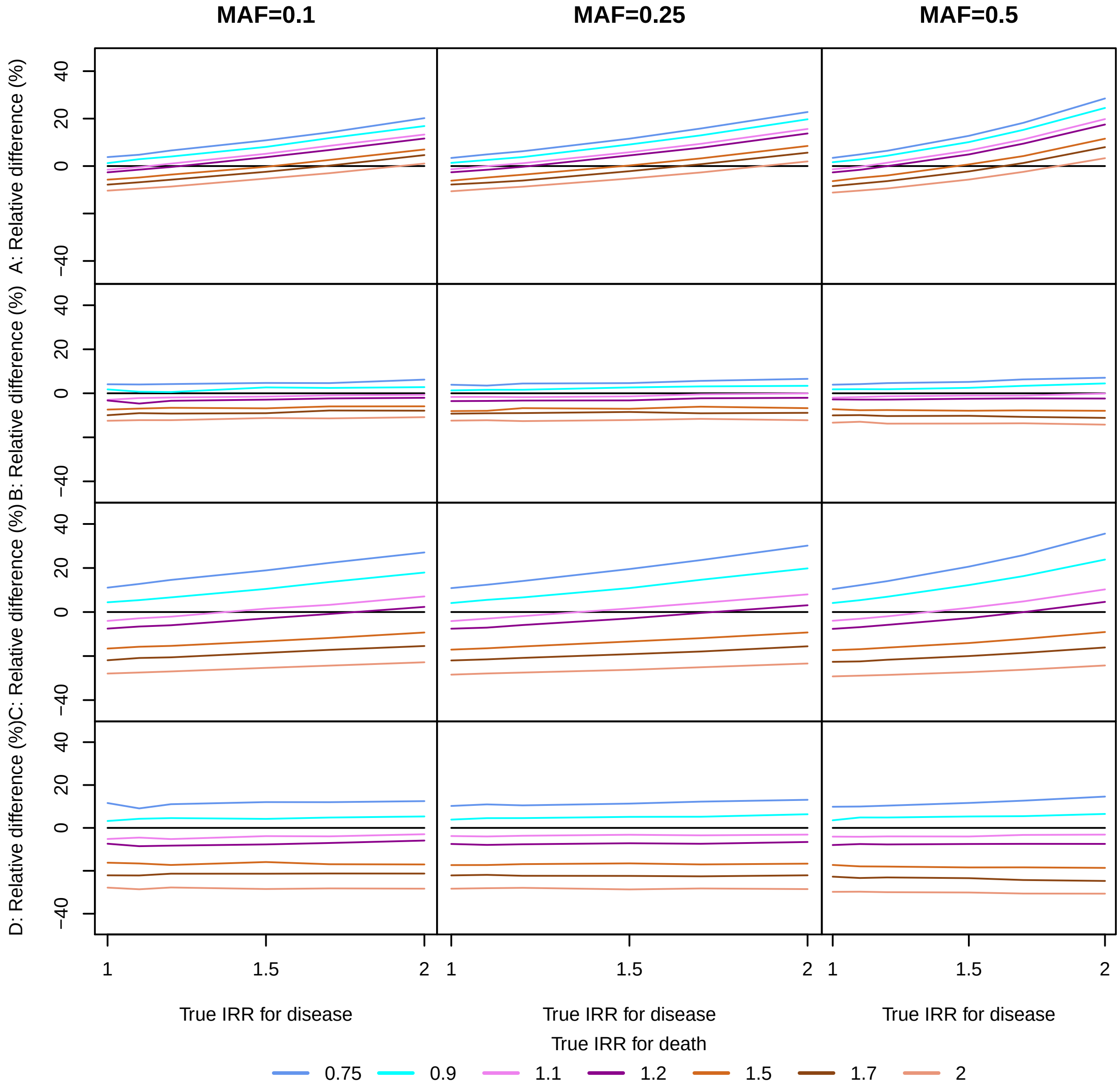

Figure S6: The relative differences in estimates. Four scenarios are displayed as in Figure 3 and Figure S1. Each subfigure presents the relative differences (%) in estimates of the association between the number of minor alleles and disease for different values of the IRR of death and the MAF. The relative difference is the difference proportional to the true IRR for disease. The different colours indicate different values for the IRR of death. The black solid horizontal line indicates no bias.

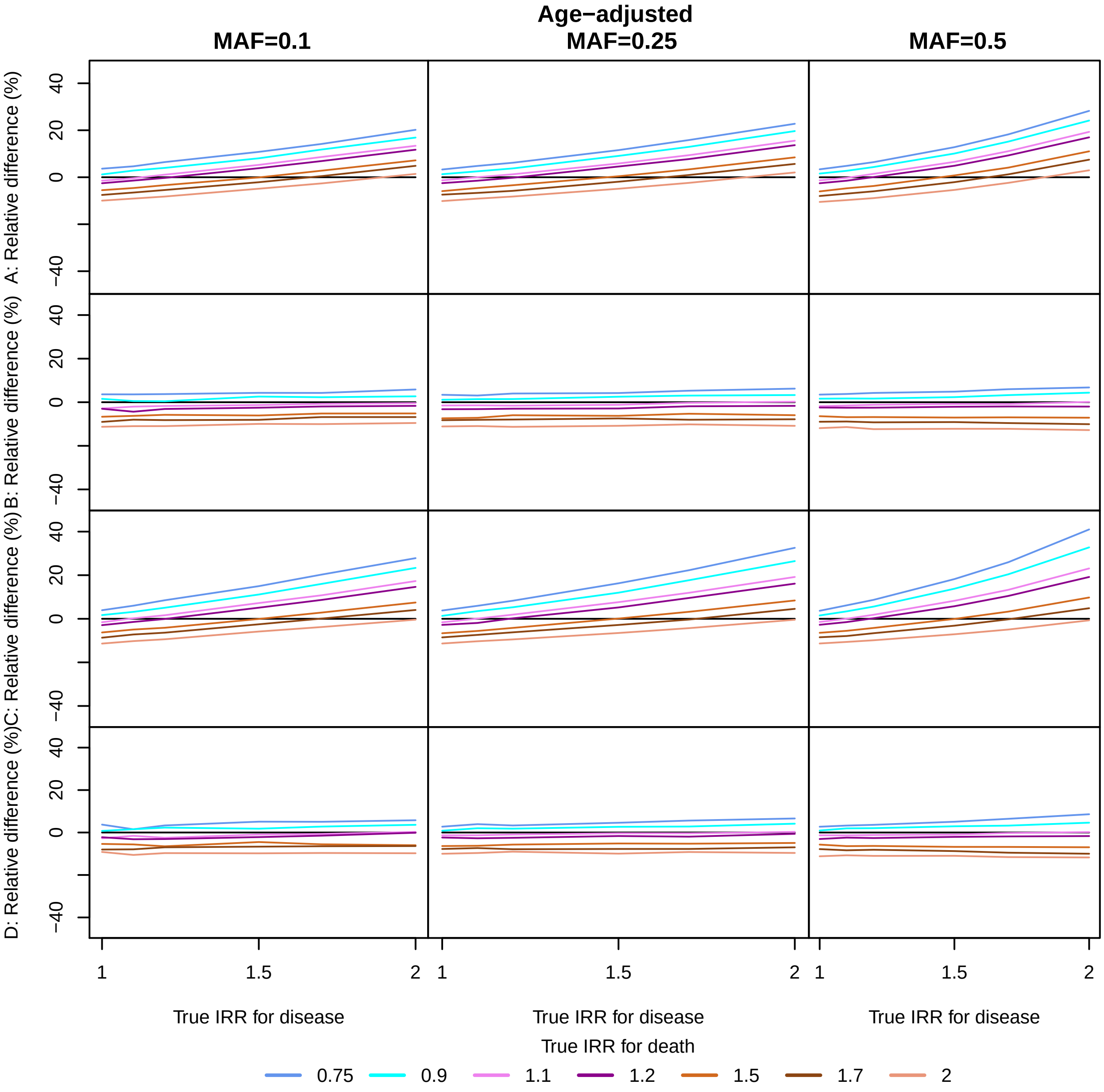

Figure S7: The relative differences in estimates when adjusting for age. Four scenarios are displayed as in Figure 3 and Figure S1. Each subfigure presents the relative differences (%) in estimates of the association between the number of minor alleles and disease for different values of the IRR of death and the MAF (adjusted for age using a linear trend). The relative difference is the difference proportional to the true IRR for disease. The different colours indicate different values for the IRR of death. The black solid horizontal line indicates no bias.

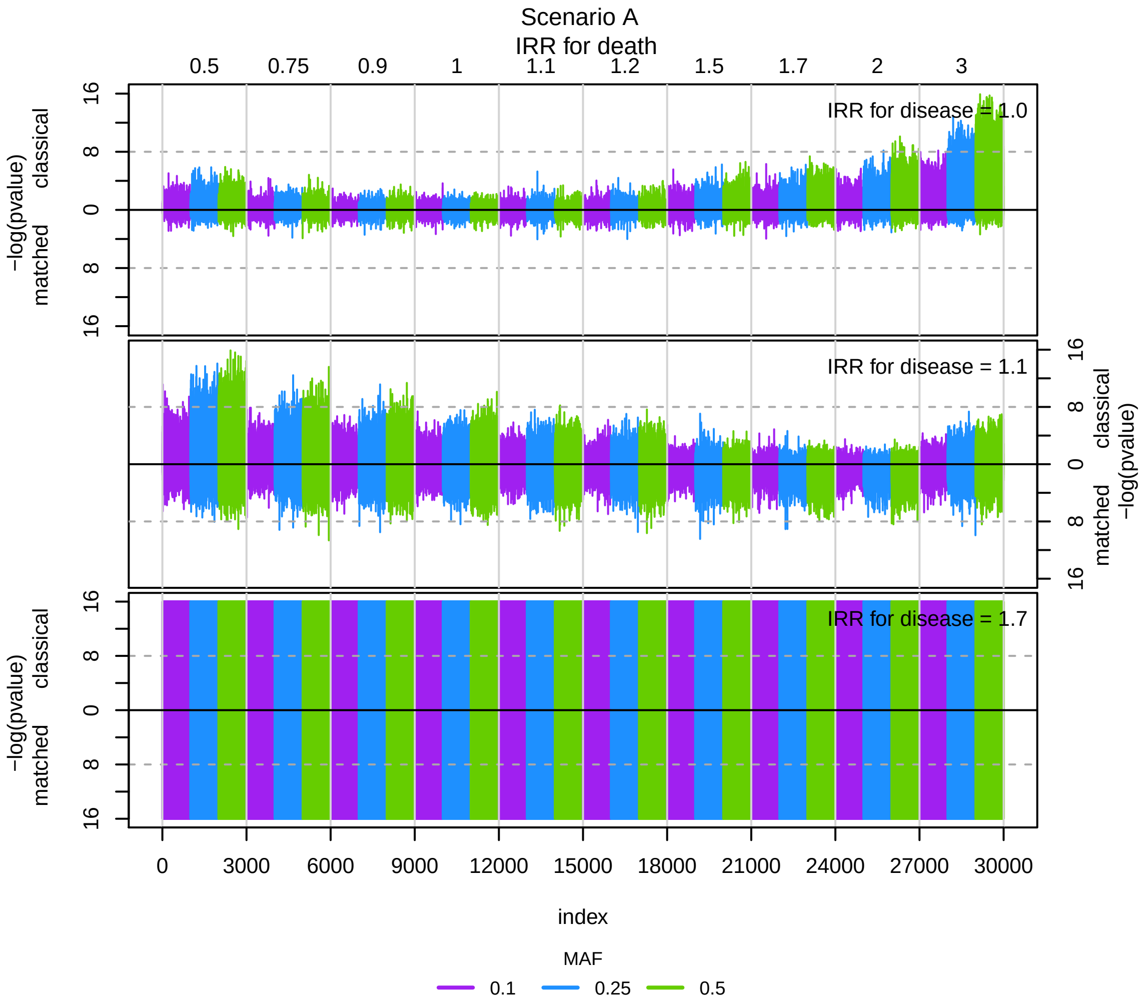

Figure S8: The changes in p-values. The supplementary figures represents scenario A (se Figure 5). The three subfigures represent the IRR for disease equal to 1.0, 1.1 and 1.7, respectively. Each subfigure is two Manhattan plots consistent of log transformed p-values from all 30,000 simulations. The log transformed p-values from the classical case-control study are found above the solid black line at 0, and the log transformed p-values from the incidence density sampling are found below the solid black line. The different colours indicate a parameter change in the MAF, and the light grey vertical lines indicate the change in the IRR of death. The horizontal dashed lines indicate the genome-wide significance level of 510^{-8}. Values larger than 510^{-16} are truncated.

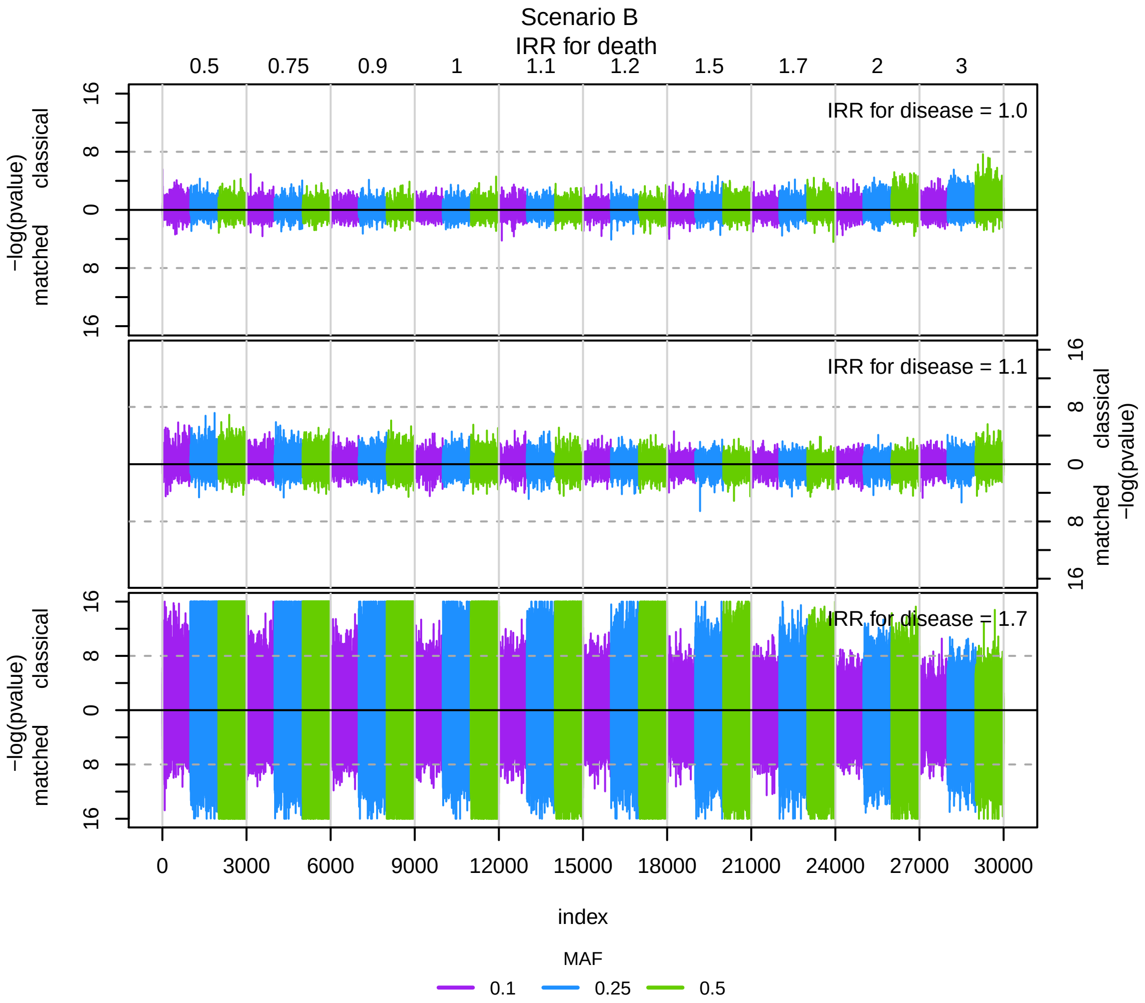

Figure S9: The changes in p-values. The supplementary figures represents scenario B (se Figure 5). The three subfigures represent the IRR for disease equal to 1.0, 1.1 and 1.7, respectively. Each subfigure is two Manhattan plots consistent of log transformed p-values from all 30,000 simulations. The log transformed p-values from the classical case-control study are found above the solid black line at 0, and the log transformed p-values from the incidence density sampling are found below the solid black line. The different colours indicate a parameter change in the MAF, and the light grey vertical lines indicate the change in the IRR of death. The horizontal dashed lines indicate the genome-wide significance level of 510^{-8}. Values larger than 510^{-16} are truncated.

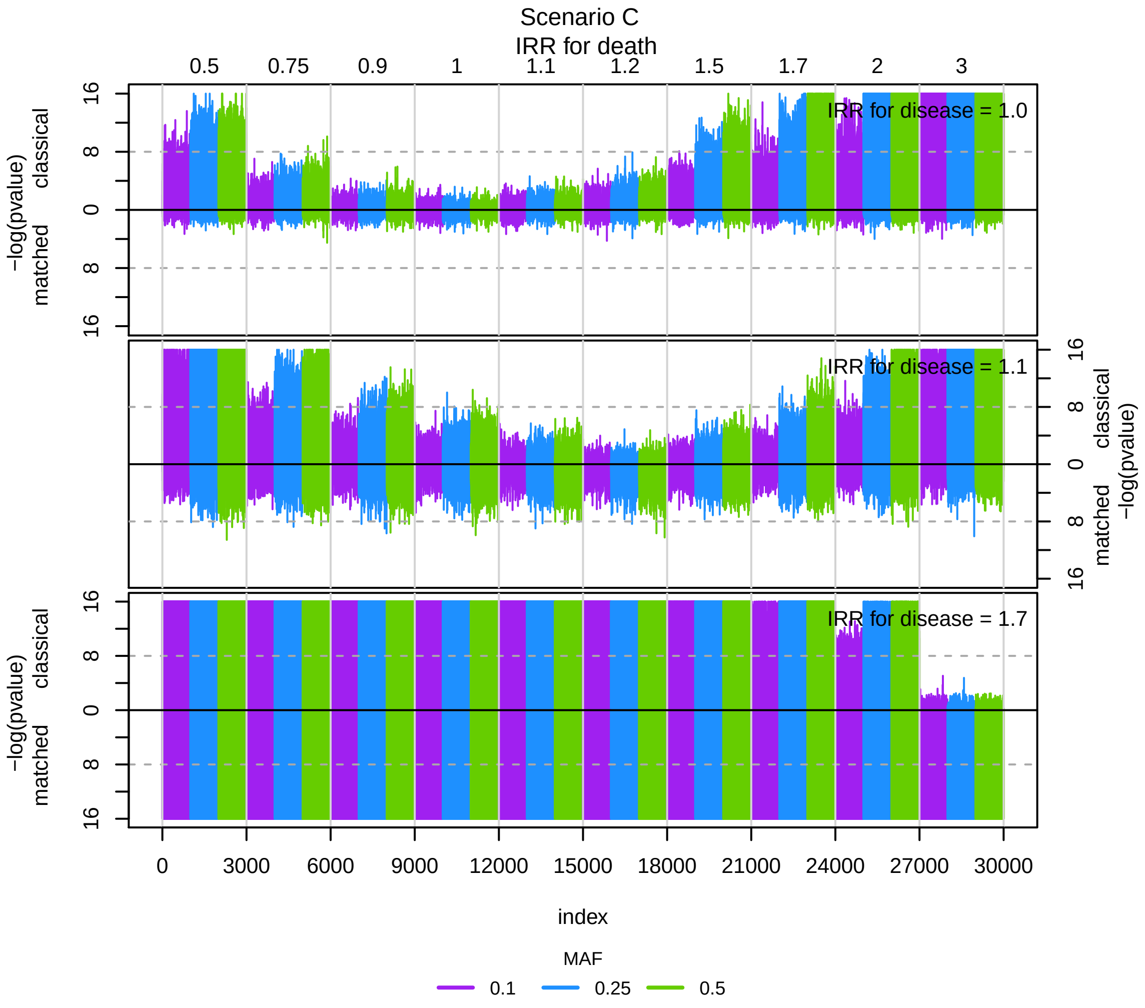

Figure S10: The changes in p-values. The supplementary figures represents scenario C (se Figure 5). The three subfigures represent the IRR for disease equal to 1.0, 1.1 and 1.7, respectively. Each subfigure is two Manhattan plots consistent of log transformed p-values from all 30,000 simulations. The log transformed p-values from the classical case-control study are found above the solid black line at 0, and the log transformed p-values from the incidence density sampling are found below the solid black line. The different colours indicate a parameter change in the MAF, and the light grey vertical lines indicate the change in the IRR of death. The horizontal dashed lines indicate the genome-wide significance level of 510^{-8}. Values larger than 510^{-16} are truncated.
